# Genetic and Epigenetic Factors in Gestational Diabetes Mellitus Pathology

**DOI:** 10.3390/ijms242316619

**Published:** 2023-11-22

**Authors:** Łukasz Ustianowski, Jakub Udzik, Joanna Szostak, Anna Gorący, Klaudia Ustianowska, Andrzej Pawlik

**Affiliations:** 1 Department of Physiology, Pomeranian Medical University, 70-111 Szczecin, Poland; l.ustianowski@gmail.com (Ł.U.); jakubudzik@wp.pl (J.U.); ustianowska.k@gmail.com (K.U.); 2 Department of Cardiac Surgery, Pomeranian Medical University, 70-111 Szczecin, Poland; 3 Department of Experimental and Clinical Pharmacology, Pomeranian Medical University, 70-111 Szczecin, Poland; joannaszostak99@gmail.com; 4 Department of Clinical and Molecular Biochemistry, Pomeranian Medical University, 70-111 Szczecin, Poland; ania.goracy@gmail.com

**Keywords:** gestational diabetes, genetics, epigenetics

## Abstract

Gestational diabetes (GDM) is the carbohydrate intolerance occurring during pregnancy. The risk factors of GDM include obesity, advanced maternal age, polycystic ovary syndrome, multigravidity, a sedentary lifestyle, and pre-existing hypertension. Additionally, complex genetic and epigenetic processes are also believed to play a crucial role in the development of GDM. In this narrative review, we discuss the role of genetic and epigenetic factors in gestational diabetes mellitus pathogenesis.

## 1. Introduction

Diabetes mellitus (DM) is a heterogeneous group of endocrine diseases that share hyperglycemia as a common characteristic [1]. Gestational diabetes mellitus (GDM) is a DM subtype manifesting during pregnancy. It may affect between 2 and 7% of pregnancies [2]. The prevalence of GDM has increased over the last two decades. Its diagnostic criteria differ between countries or even between different scientific organizations [3]. Example diagnostic criteria from the American Diabetes Association (ADA) are fasting glucose level ≥ 5.1 mmol/l or glucose concentration ≥ 10 mmol/l in the 60th minute of OGTT or ≥ 8.5 mmol/l in the 120th minute of OGTT [1]. A fasting glucose level of ≥ 7.0 mmol/l or glycemia ≥ 11.1 mmol/l in the 120th minute of OGTT is diagnostic of DM during pregnancy rather than GDM [4]. Screening for GDM may occur during the first visit to a gynecologist during pregnancy. If undiagnosed or untreated, GDM can lead to pre-eclampsia, macrosomia, and polyhydramnios [5]. Moreover, individuals previously diagnosed with GDM are prone to a diagnosis of DM type 2 after delivery [6].

**Figure 1:**
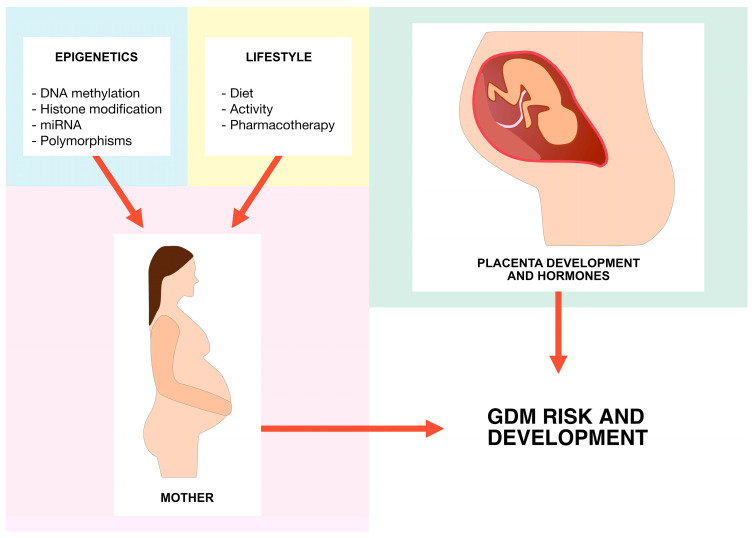
GDM risk factors.

**Figure 2:**
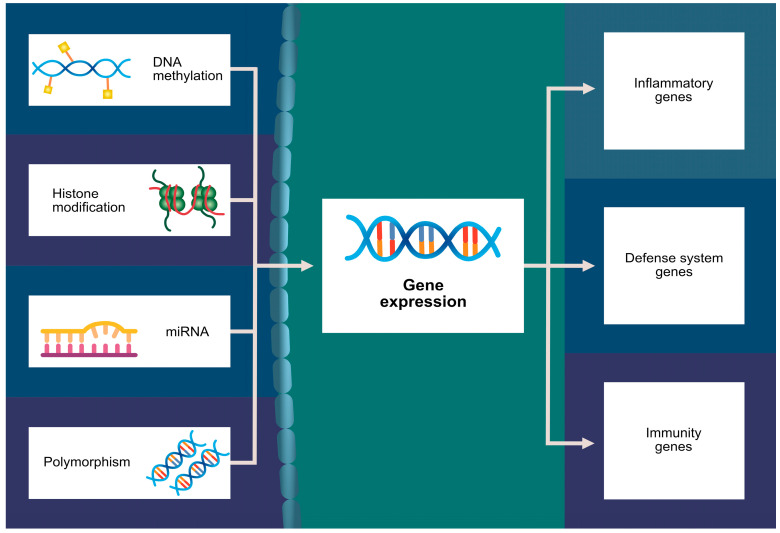
Potential epigenetic alterations participating in GDM pathogenesis.

While multiple risk factors for GDM (i.e., body mass index (BMI) ≥ 27 kg/m^2^, advanced maternal age, polycystic ovary syndrome, multigravidity, a sedentary lifestyle, and pre-existing hypertension; Figure 1) have already been identified, complex genetic and epigenetic processes are also believed to play a crucial role in the development of GDM [7,8,9]. It is considered to be the most common metabolic complication of pregnancy [10]. Different epigenetic changes like DNA methylation, histone modifications, or microRNA (miRNA; miR) gene silencing have already been confirmed in GDM patients (Figure 2) [11]. Increased concentration of hormones antagonistic to insulin is one of the suggested mechanisms in GDM development [2]. Some individuals might be genetically predisposed due to impairment of pancreatic islet β-cell function [12,13].

## 2. MODY

Maturity-onset diabetes in the young (MODY) is a type of hereditary DM responsible for 5% of all diabetes cases [14]. A monogenic mutation disrupts the physiological response to changes in glucose levels, with subsequent metabolic changes typical of diabetes. It usually manifests before 25 years of age and is primarily caused by pancreatic β-cell dysfunction. Many studies have investigated contributory genetic factors. To date, 14 MODY mutations have been found (Table 1). Their prevalence varies widely depending on many factors, but the most common MODY types and gene mutations are the following:

- MODY 3: hepatocyte nuclear factor 1 alpha (*HNF1A*)
- MODY 1: hepatocyte nuclear factor 4 alpha (*HNF4A*)
- MODY 2: glucokinase (*GCK*)
- MODY 5: hepatocyte nuclear factor 1 beta (*HNF1B*)

These four genes account for around 70 to 90% of all MODY types, depending on the sample size and region of study [14,15,16,17,18]. However, there are more mutations also associated with MODY, responsible for many metabolic actions, such as ATP binding cassette subfamily C member 8 (*ABCC8*), B-lymphocyte kinase (*BLK*), carboxyl ester lipase (*CEL*), neurogenic differentiation 1 (*NEURO1*), paired box 4 (*PAX4*), pancreatic duodenal homeobox (*PDX*), and others. MODY can be first diagnosed during pregnancy and it is speculated that it may account for 5% of all GDM cases detected by routine screening [17].

### 2.1. MODY 1

*HNF4A* regulates the transcription of HNF1A. Patients with MODY1 are at a higher risk of macrosomia and high birth weight, with the mean birth weight being 790 g higher than that of healthy babies [19]. Also, infants with MODY1 born to MODY1-affected mothers have a higher risk of macrosomia than those with affected fathers. As a result, rigorous ultrasound monitoring during the third trimester is recommended [20,21]. Also, after the birth, the infant should be monitored for at least 48 h for possible hypoglycemia. As for the therapeutics, no treatments have been shown to improve fetal outcomes. For women, it is recommended to start insulin before conception. Another possibility is to use sulfonylureas (SU) under tight maternal glycemic control and then change to insulin in the second trimester. However, SU can cross the placental barrier and, as such, its use during the first trimester is often debated [19].

### 2.2. MODY 2

GCK is an enzyme crucial for glucose metabolism and energy production within tissues [22]. Mutations in the *GCK* gene are associated with MODY 2, which accounts for 0.4 to 1% of GDM cases [23]. The highest rate of GCK expression is observed in the heart, placenta, brain, and liver tissue. During pregnancy, GCK concentration and activity rise in maternal pancreatic β-cells as part of the adaptation process to decrease insulin sensitivity [24]. GCK functions as a glucose sensor for the β-cell, and mutations in GCK increase the threshold for insulin release [19]. MODY 2 clinically results in raised fasting glucose levels from birth [25]. The investigations conducted so far indicate that changes in the methylation of the *GCK* gene are a risk factor for type 2 diabetes [26,27]. Chen et al. describe a pathway involving insufficient demethylation of the *GCK* gene, by which maternal glucose intolerance can be inherited by their offspring [28]. Stride et al. reported that the use of oral hypoglycemic agents or insulin did not significantly change overall glycemic control [29]. It is recommended that MODY 2 mothers strictly monitor their glucose levels during pregnancy and insulin should be administered if needed [19].

### 2.3. MODY 3

HNF1A is a protein crucial to developing β-cells in the pancreas. *HNF1A* gene mutation can decrease insulin production, resulting in higher glucose levels. MODY 3 is the most common form of MODY [30], with several single nucleotide polymorphisms (SNPs) identified [17]. Bellanné-Chantelot et al. screened a group of patients for an association between SNP mutations and the age of onset of DM. They found that 83% of mutations in the *HNF1A* gene were located in exons 1 to 6, affecting three studied gene isoforms [30]. Missense mutations were found in 74% of patients with MODY 3, and 64% had truncated mutations of the *HNF1A* gene. The number of missense mutations affecting *HFN1A* isoforms was inversely proportional to the age of onset [30]. During pregnancy, MODY 3 is sometimes related to neonatal hyperinsulinemic hypoglycemia and a lower threshold for renal excretion of glucose. Stride et al. showed that combined screening for β-cell deficiency and renal dysfunction could be used to screen children as the presence of a glycosuria post-glucose load occurs in all mutation carriers with MODY 3 with a peak glucose level during OGTT of over 8.4 mmol/L [31]. MODY 3 is relatively well controlled with SU. However, during pregnancy, it should be treated with insulin. Regarding fetal monitoring, MODY 3 should be treated as pre-existing diabetes [19]. Fetal inheritance of MODY 3 is not related to increased birth weight or incidence of hypoglycemia. Recent recommendations focus on balancing considerations of uncontrolled hypoglycemia with the risk of fetal macrosomia [19].

### 2.4. MODY 4

*PDX1*, also known as insulin promoter factor 1 (IPF1), is a gene expressed predominantly in pancreatic β-cells [32]. Decreased *PDX1* expression is associated with an increased risk of the development of type 2 diabetes [32]. During pregnancy, *PDX1* is vital to both the maternal pancreatic β-cells’ proliferation and embryonal pancreatic development [33]. Wang et al. demonstrated decreased *PDX1* expression within the placentas of women with GDM, although hypermethylation was not the leading epigenetic mechanism in that population [34]. Kaimala et al. suggest that the *PDX1* gene can be suppressed by the deacetylation of the H4K8 and H4K16 histones, leading to GDM [35]. Studies in animal models have found that exposure to bisphenol A (BPA) during pregnancy impacts the acetylation and methylation of histones, resulting in *PDX1* downregulation and the development of GDM [36].

### 2.5. MODY 5

HNF1B-MODY patients have lower insulin levels compared to healthy individuals. Therefore, MODY 5 typically requires insulin therapy for glycemic control, with no recommendations for SU use, especially during pregnancy [37,38]. Individuals with HNF1B-MODY born to HNF1B-MODY-affected mothers had higher birth weights, while those with HNF1B-MODY and unaffected mothers had a 69% incidence of being small for gestational age (SGA) [39].

### 2.6. MODY 12

ATP binding cassette subfamily C member 8 (*ABCC8*), also known as sulfonylurea receptor 1 (*SUR1*), is a gene that encodes a subunit of the ATP-dependent potassium channel within the pancreatic β-cells [40]. Studies in animal models have indicated that *ABCC8* hypermethylation is responsible for hereditary glucose intolerance [40,41]. A new review by Zhu et al. summarizes the evidence of adverse chemical exposure altering the individual predisposition to diabetes. They show that induced diabetic susceptibility can also be transmitted to the next generations [41]. Activating mutations are responsible for diabetes from the early neonatal stages, while inactivating mutations cause congenital hyperinsulinism [42]. MODY 12 presents similarly to HNF1A and HNF4A MODY and, therefore, reacts well to SU treatment [42]. Thus far, there have been no reports of epigenetic modifications to *ABCC8* that would increase the risk of GDM.

### 2.7. MODY 13

The potassium inwardly-rectifying channel, subfamily J, member 11 (*KCNJ11*) gene encodes the Kir6.2 protein, a structural element of the ATP-dependent potassium channel within pancreatic β-cells [43]. Mutations within this gene may lead to abnormalities in insulin secretion and the development of diabetes, especially in neonates [44,45]. As of yet, there is no indication that epigenetic mechanisms related to *KCNJ11* play a role in the development of GDM. Higher levels of methylation can mimic the suppression of the *KCNJ11* gene and are associated with an increased risk of metabolic syndrome [46,47].

## 3. Adiponectin S, Leptin, and Interleukins

Epigenetic alterations are thought to have a connection with obesity and other metabolic diseases. Previous studies have reported that the methylation frequency of leptin (LEP) and adiponectin (ADIPOQ) promoters may contribute to the development of metabolic syndrome [48]. A review by Xu et al. shows the placental secretion of various hormones in pregnant women. They disrupt maternal insulin resistance thresholds and lead to hyperglycemia. DNA methylation of those molecules and their pathway-related genes are also related to the pathogenesis of GDM. Adiponectin is a hormone secreted by adipocytes, with presumed insulin-sensitizing, anti-inflammatory, and anti-atherosclerotic functions [49]. Bouchard et al. have shown that lower levels of DNA methylation in the ADIPOQ promoter on the fetal side of the placenta were associated with higher maternal glycemic levels during the second trimester of pregnancy. Additionally, during the second and third trimesters of pregnancy, lower DNA methylation levels on the maternal side of the placenta were also connected with maternal insulin resistance [50]. DNA methylation of the *ADIPOQ* gene locus was related to higher circulating maternal adiponectin levels during pregnancy and after delivery. Furthermore, it has been demonstrated that even intermediate glucose intolerance is correlated with the *ADIPOQ* DNA methylation profile [50]. Overall, these results suggest that epigenetic changes around *ADIPOQ* are possible mechanisms in the fetal programming of metabolic disorders in adults.

Leptin is secreted in paracrine and endocrine manners into the blood by adipocytes. Insulin triggers leptin secretion and activates the *LEP* gene. The significance of insulin in regulating leptin levels and signaling shows the crucial role of leptin in obesity-induced insulin resistance. Lesseur et al. determined that higher *LEP* gene methylation in the placentas occurs in patients with GDM and pre-pregnancy obesity. Their research indicates that the maternal metabolic state before and throughout pregnancy can influence the placental DNA methylation profile at birth. Subsequently, it may contribute to the metabolic programming of obesity and related diseases in children [51]. Bouchard et al. in their research studied DNA methylation levels in the leptin gene in placentas exposed to IGT during pregnancy compared to women with normal glucose tolerance (NGT). In the IGT group, glucose levels were positively correlated with methylation on the maternal side. On the contrary, the correlation was negative on the fetal side [52]. Leptin mRNA levels were negatively correlated with *LEP* promoter methylation on both sides of the placenta. These results imply that dysregulation of the DNA methylation profile, particularly IGT-related DNA methylation changes, may contribute to long-term consequences associated with fetal programming, such as an increased risk of developing obesity and type 2 diabetes [52]. Epigenetic changes both in the *LEP* gene and the *ADIPOQ* gene provide new insights that can help to explain the mechanisms of fetal programming. This may help to determine its health effects and improve diagnostics and therapies.

Earlier studies evaluated blood samples from pregnant women with GDM to determine the methylation profile of genes involved in the inflammation process. Dłuski et al. showed that various other methylation profiles change in pregnant women, including inflammatory processes, neuronal development, and even cellular pathways [53]. Halvatsiotis et al. showed that only the *ATF2* gene was hypermethylated in GDM patients. On the contrary, the genes encoding the interleukins and interleukin receptors, such as *IL4R*, *IL6R*, *IL17RA*, *IL12A*, *IL13*, and *IL10RA,* were found to only be hypomethylated in pregnant women with GDM [54]. IL-10 is an anti-inflammatory cytokine produced by T cells, B cells, and macrophages. It both stimulates and suppresses the immune response. Additionally, it participates in cell activation, proliferation, and differentiation [49]. It has been stated that the IL-10 plasma level is lower in GDM patients than in people without GDM. There is a correlation between low IL-10 levels and excessive insulin resistance. This may suggest that low IL-10 concentrations contribute to insulin resistance in GDM patients [55]. The typical level of methylation of the IL-10 gene in a GDM group was lower than in maternal blood samples from a non-GDM group [56], supporting the hypothesis that epigenetic modifications are related to the etiology of GDM. Qiu et al. showed the upregulated expression of miR-518d in placentas affected by GDM compared to a healthy pregnancy group. Further, increased mRNA levels of nuclear factor-kappa B (NF-κB), cytochrome C oxidase subunit II (COX-2), TNF-α, IL-1β, IL-6, and decreased mRNA levels of peroxisome proliferator-activated receptor α (PPARα) were presented in placentas in women with GDM. In the study, *miR-518d* was believed to promote the mRNA expression of COX-2, TNF-α, IL-1β, and IL-6, but PPARα was negatively regulated by it. This may imply an association between the development of GDM and inflammatory response in placentas regulated by *miR-518d* [57]. However, it would be premature to assume that epigenetic changes predate GDM. Investigating epigenetic changes in other genes involved in energy balance, glycemic regulation, and insulin resistance pathways will be essential in establishing the causality of GDM. The determination of molecular mechanisms and genetic involvement in the fetal programming of energy metabolism will assist in understanding the pathophysiological processes leading to metabolic disorders. However, Valenzia-Ortega et al. point to the fact that specific causality is yet to be established, and more studies should focus on exploring changes in gene expression [58].

## 4. The β3-Adrenergic Receptor (ADRB3)

ADRB3 is a catecholamine-stimulated receptor found on cell walls and is expressed in many tissues including skeletal muscle and pancreatic β-cells [59,60]. However, it is mainly found in adipose tissue, where it mediates thermogenesis and lipolysis. It is bound by noradrenaline to induce metabolic changes [61].

ADRB3 is coded for on the short arm of chromosome 8. A connection between *ADRB3* polymorphisms and metabolic syndrome was reported in 2004 by Parikh and Groop [62]. A *Trp64Arg* SNP polymorphism was found to influence the risk of insulin resistance, abdominal obesity, and the early onset of type 2 diabetes [63,64]. Further studies showed that this SNP variant influences insulin secretion, both in vivo and in vitro [65,66]. Additionally, this polymorphism has been associated with increased weight gain and higher glucose and insulin levels during pregnancy [67]. A study by Festa et al. associated *Trp64Arg* with increased glucose levels during pregnancy [68]. On the contrary, a study by Alevizaki et al. showed no association between *Trp64Arg* and GDM [69]. As SNPs have been more widely investigated, other *ADR3B* SNPs have been found that are associated with an increased risk of GDM. A metanalysis by Zhang et al. focused on the relationship of 10 genes and their SNPs to GDM. *ADRB3* was found to have no statistical correlation with GDM from the pooled results of five studies [70].

## 5. Insulin Receptor

The insulin receptor (INSR) is a ligand-activated transmembrane signaling protein that belongs to the tyrosine kinase group [71]. The INSR is responsible for metabolism regulation [71]. Mutations in INSR have been observed in patients with extreme insulin resistance [72]. The hypermethylation of cytosine-phosphate-guanine (CpG) dinucleotides in the promoter of INSR is found in women with GDM [73] compared to normoglycemic pregnant women [74]. Furthermore, levels of the INSR protein are lower in pregnant women without GDM than in women with GDM [72]. Insulin-like growth factor 2 (IGF2) is a part of the insulin group and has an influence on metabolic disorder development [75]. Another study focused on the association between a higher risk of GDM occurrence and polymorphisms in the restriction fragment length of INSR and IGF2 and found that Caucasian women with polymorphisms in INSR as well as in IGF2 had an increased risk of GDM [76].

### 5.1. Insulin Receptor Substrate 1

Hormonal control of metabolism is provided by insulin receptor substrate 1 (IRS1), one of the main targets of the insulin receptor tyrosine kinase enzyme [77]. Patients with diabetes and insulin resistance are differentiated by dysfunction of IRS-dependent signaling in their tissues [77]. Insulin resistance observed during pregnancy may be related to an increase in maternal and placental hormones [78] including prolactin, progesterone, estrogen, placental growth hormone (hPGH), and human placental lactogen (hPL) [78]. Each hormone contributes to insulin resistance in pregnant women through different mechanisms [78]. Progesterone is responsible for the suppression of IRS-1 expression [78]. Increased levels of estradiol lead to insulin resistance by the serine phosphorylation of IRS-1, which is a result of c-Jun N-terminal kinase (JNK) activation mediated by estrogen receptors (ER) [79]. Moreover, the influence of hPGH on insulin resistance is associated with increased expression of the p85-regulatory unit of PI3-kinase (PI3K). This causes a decrease in RS-1-associated PI3K activity [78]. hPL is considered to be the primary insulin resistance hormone that leads to reduced phosphorylation of IRS-1 [80].

### 5.2. Insulin-Sensitive Glucose Transporter Protein 4/Solute Carrier Family 2 Member 4

The insulin-sensitive glucose transporter protein 4 (GLUT4), also known as the solute carrier family 2 member 4 (SLC2A4), is encoded by the *SLC2A4* gene [81,82]. GLUT4 protein expression is regulated by estrogens, which may explain why those hormones have an impact on insulin resistance progression [81]. The SLC2A4 protein is responsible for postprandial glycemic control [83] due to its action as an insulin-dependent glucose transporter [82]. A study by Li and Zhang showed that decreased SLC2A4 expression was observed both in blood samples and pancreatic cell lines collected from type 2 diabetes patients [82]. Insulin resistance in women with GDM can also be a result of imperfect SLC2A4 translocation [72]. GLUT4 is normally translocated to the plasma membrane due to insulin stimulus [83]. Moreover, women who suffer from GDM are characterized by a lower level of SLC2A4 protein in their adipose tissue in comparison to healthy pregnant women [84].

## 6. Plasma Cell Membrane Glycoprotein 1

The plasma cell membrane glycoprotein 1 (PC-1) is encoded by the ectonucleotide pyrophosphate phosphodiesterase-1 (*ENPP1*) gene, which is expressed in adipose tissue and skeletal muscle [72]. *ENPP1* is responsible for reducing the tyrosine kinase activity of insulin receptors [84], which makes it a probable insulin resistance gene [83]. Furthermore, tissue ENPP1 protein levels have been found to be significantly lower in pregnant women who are not diagnosed with GDM than in women with GDM [72]. Moreover, SNPs in the *ENPP1* gene have been found to play a role in positive OGTTs [85]. There is also a higher risk of diabetes type 2 in obese people who have been found to have SNPs in the *ENPP1* gene, as well as an association with obesity and an increased chance of GDM [85].

## 7. Calpain 10

Calpain 10 (CAPN10) is a cysteine protease dependent on calcium ions encoded by the *CAPN10* gene in the human genome. Calpains are activated by calcium ion influx and they then catalyze the controlled proteolysis of targeted proteins [86]. The calpain family has been implicated in a variety of diseases including Alzheimer’s disease, ischemic stroke, and limb–girdle muscular dystrophy 2A [87,88,89]. There has been a focus of research on this after gene scanning was conducted in search of diabetes predisposition factors. The *CAPN10* gene was the first to be identified as a predisposing factor by the positional cloning approach [90]. Despite extensive investigation of calpain in the pathophysiology of diabetes, its exact properties remain unknown. It takes part in many metabolic pathways, including cell cycle regulation, apoptosis, and signal transduction [91]. Laske et al. showed that upregulation of *CAPN10* is present in Alzheimer-type diseases, where it increases the accumulation of β–amyloid peptides. It leads to hyperphosphorylation in the central nervous system and the degeneration of neurologic functions [92]. *CAPN10* gene polymorphisms are associated with the risk of developing type 2 DM. A study by Wu and Car [93] focused on two SNPs found in the *CAPN10* gene: SNP43 (G/A) and SNP63 (C/T), and their relation to cerebral small vessel disease. Patients with SNP43 had an increased risk of cognitive impairment in cerebral small vessel disease, type 2 diabetes, and elevated fasting serum insulin. In another study, Perez-Martinez et al. analyzed 452 subjects with metabolic syndrome (MS) for several MS-related factors and five *CAPN10* polymorphisms. They found that the rs2953171 *CAPN10* polymorphism may influence insulin sensitivity. It was found to interact with plasma fatty acid composition in MS patients with higher fasting insulin and HOMA-IR values [94]. Another approach to investigating the epigenetics of *CAPN10* is to look for complementary DNA synthesized from mRNA used to express specific proteins. Ono et al. [95] found that the *CAPN10* cDNA transcript is subject to cryptic splicing. This led to unexpected protein products being expressed, and the team analyzed two *CAPN10* isoforms. The two isoforms had different substrate proteolysis and potential cell functions, demonstrating that, recombinantly expressed, CAPN10 proteins may express different cell actions, with further research needed to study alternative expression routes [95].

## 8. Histone Modification

Histones are proteins that wrap the DNA around themselves and provide structural support for DNA. Condensing DNA into chromatin enables long DNA strands to fit inside the nucleus. Histones can be modified by several enzymatic reactions that affect their structure with a subsequent chromatin structure. By unwrapping the specific DNA region, they enable gene expression, and by tightening the chromatin structure, they suppress gene expression. Those changes control cell metabolism. Therefore, histone modification can alter all metabolic pathways, with potential clinical consequences.

Görisch et al. [96] showed that histone acetylation increased chromatin accessibility, thus creating an active euchromatin state and increasing gene expression. Additionally, Kimura has shown that transcription sites are marked with trimethylated H3K4 histones and acetylated H3K27 histones, and gene repression is achieved by the trimethylation of H3K9 histones and H3K27 histones [97]. Histone modification is responsible for the potential increased risk of GDM. Argreaves and deacetylases were found to be responsible for modifying the histone, with subsequent under-expression of GLUT4, in a study by McGee [98].

MODY 4 is associated with impaired PDX1 function, which is responsible for pancreas and β-cell development. Histone acetylation, combined with reactive oxygen species, has been shown to result in suppressed PDX1 expression and potentially be correlated with the risk of GDM by Fernández-Morera et al. [99]. Histone modification and the risk of GDM were also investigated by Michalczyk et al. [100], who found that specific histone methylation patterns could be a basis for predicting the risk of GDM. The study by Hepp et al. analyzed histone modifications in 40 control and 40 GDM placentas. They demonstrated that H3K9ac expression was downregulated in GDM ones, especially in syncytiotrophoblast, EVT, and fetal endothelial cells. H3K9ac is crucial in modifying transcription activity, especially throughout intrauterine development, synzytialisation, and angiogenesis. This indicates that the downregulation of H3K9ac in GDM may lead to an insufficient capacity for gene expression and, subsequently, to the development of fetal complications such as organ immaturity [101]. However, more data are needed to evaluate the effect of histone modification on GDM risk and explore potential treatment options.

## 9. miRNA

miRNAs are non-coding RNAs used in post-transcriptional gene expression control. They interact with messenger RNA to degrade or inhibit their translation [102]. As they can control gene expression, their impact on epigenetic changes has been studied to assess their role in developing diseases, including GDM. Zhao et al. [103] focused on three miRNAs found in pregnant women with GDM: MiR-29a, miR-222, and miR-132 all had decreased plasma concentrations in the control group. Those findings were later supported by studies that found 12 miRNAs that were over-expressed in GDM women and were found to relate to glucose and insulin metabolism. They disrupted the expression of mitogen-activated protein kinase (*MAPK*) and *IRS* genes. This resulted in impaired signaling pathways within cells [104].

A meta-analysis of the association between miRNA and GDM was performed by da Silva et al. [105] and identified 82 GDM-related miRNAs, with 4 dysregulated miRNAs being the most frequently cited: miR-16, miR-330, miR-20a, and miR-222. They are responsible for glucose and insulin metabolism and controlling the metabolic pathways of pancreatic β-cells. MiR-16, when upregulated, was observed to downregulate the insulin signaling pathway, with possible resulting chronic hyperglycemia [106]. The upregulation of miR-330 is associated with β-cell impairment by altering their proliferation and growth [107]. MiR20a is responsible for regulating cell metabolic pathways responsible for glucose homeostasis. Therefore, its dysregulation results in hyperglycemia and it may be a possible biomarker for GDM [108,109]. MiR-222 is found in maternal plasma in the highest concentration during weeks 24 to 28 of pregnancy. It is produced by the placenta and is responsible for estrogen receptor-α expression in estrogen-induced insulin resistance. A study by Filardi et al. showed that dysregulated miRNA levels were correlated with increased fasting plasma glucose and increased birth weight [110,111,112]. Another study by Dong et al. focused on potential neural tube defects in neural stem cells. They were cultured in a normal or high glucose medium, with subsequent measures of miR-200c levels. It was shown that the high glucose medium caused an upregulation of miR-200c, resulting in neural stem cell damage, potentially providing a pathway for neural tube defects found in the offspring of mothers with GDM [113].

A new study suggests that long non-coding RNA Meg3 found in the liver may contribute to impaired glucose metabolism. A novel study by Yang et al. uses an animal model to mimic GDM and its effects on offspring. They showed that intrauterine exposure to hyperglycemia impairs pregenital glucose and insulin resistance. The expression of Meg3 in the liver was increased, leading to significant differences in PPAR signaling pathways. They suggest new insights into new possible pathways of DM in the offspring of GDM mothers [114].

As more and more data are being published about miRNAs, miRNA panels are being proposed as a viable option for the risk assessment of different illnesses. A study by Mitra et al. [115] proposed the creation of race- and region-specific miRNA panels for GDM screening in large populations. This is to create forecasting strategies to lower GDM prevalence in mothers and their children. Exosomal microRNA (ExomiRs) are potential targets for understanding the pathophysiology of β-cell dysfunction in GDM. According to a study by Mitra et al., the overexpression of certain ExomiRs may help overcome insulin resistance by modifying glucose uptake. Thus, this capacity makes ExomiRs promising to be used as a clinical tool for reducing the risk of GDM and other pregnancy complications. As a result, many pregnancy complications could be avoided by the appropriate intervention [115]. However, with the high costs of miRNA detection, more research is needed to establish the predictive value of miRNA in GDM.

As more and more data are being published about miRNAs, miRNA panels are being proposed as a viable option for the risk assessment of different illnesses. A study by Mitra et al. [115] proposed the creation of race- and region-specific miRNA panels for GDM screening in large populations for the development of forecasting strategies to lower GDM risk in mothers and their children. However, with the high costs of miRNA detection, more research is needed to establish the predictive value of miRNA in GDM.

## 10. Conclusions

Gestational diabetes mellitus (GDM) is a disorder of carbohydrate metabolism that occurs in pregnant women. Underlying GDM is decreased insulin secretion by pancreatic beta cells and tissue insulin resistance. The development of GDM is influenced by a number of environmental factors, such as obesity, the woman’s age, improper diet, and a number of genetic and epigenetic factors. The development of this complication occurs when there is an interaction between environmental, genetic, and epigenetic factors. Among the most important genetic factors considered so far are genes that affect the function of pancreatic beta cells and thus insulin secretion. It is believed that among these genes, HNF1A, HNF1B, HNF4A, and GCK may be the most important. Among the rarer mutations leading to pancreatic beta-cell dysfunction are mutations within the IPFI/PDX1, KCNJII, ABCC8, CAPN10, INSR, and GLUT4/SCLA4 genes. There are also various epigenetic mechanisms, such as histone modification and non-coding RNAs, which may contribute to the onset of carbohydrate disorders in pregnant women and the development of GDM. However, it is important to remember that a number of factors can lead to GDM that disrupt pancreatic beta cell function and increase tissue insulin resistance. Previous studies have shown that many genes are associated with pancreatic beta-cell dysfunction and may predispose women to the development of GDM. However, environmental factors modulate, through epigenetics, the influence of these genes on the risk of GDM. Only the interaction between environmental, genetic, and epigenetic factors can lead to the development of this complication in pregnant women.

## Figures and Tables

**Table 1:** Mutations of MODY and GDM. GDM—gestational diabetes mellitus; MODY—maturity-onset diabetes in the young.

MODY Type	Gene	Full Name	Mutation Influence on Pathophysiology
**Most common mutations accounting for 70–90% of MODY cases**			
MODY 3	*HNF1A*	Hepatocyte nuclear factor-1 alpha	Gradual beta-cell dysfunction, reduced insulin production, and progressive hyperglycemia
MODY 1	*HNF4A*	Hepatocyte nuclear factor-4 alpha	Progressive beta-cell dysfunction, fetal macrosomia, and hyperinsulinemic hypoglycemia
MODY 2	*GCK*	Glucokinase	Disrupted glucose sensing and hyperglycemia
MODY 5	*HNF1B*	Hepatocyte nuclear factor 1B	Dysfunctional pancreatic development, suppressed cytokine signaling, and formation of kidney cyst
**MODY mutations of lower prevalence**			
MODY 4	*IPFI/PDX1*	Insulin promoter factor/pancreatic duodenal homeobox	Pancreatic agenesis, beta-cell development, and defective insulin secretion
MODY 13	*KCNJII*	Inward-rectifier potassium channel, subfamily J, member 11	Congenital hyperinsulinism
MODY 12	*ABCC8*	ATP binding cassette subfamily C member 8	Congenital hyperinsulinism, disrupted biogenesis, and insulin trafficking of KATP channels
**Other GDM mutations**			
	*CAPN10*	Calpain-10	Dysfunction of cell metabolism and signal transduction and elevated fasting glucose levels
	*ADRB3*	β3-adrenergic receptor	Decreased insulin excretion, disrupted thermogenesis, and lipolysis
	*INSR*	Insulin receptor	Disrupted metabolism of β-cell and elevated glucose levels
	*IRS1*	Insulin receptor substrate 1	Dysfunction of intracellular signaling and increased insulin resistance
	*GLUT4/SCLA4*	Insulin-sensitive glucose transporter protein 4/solute carrier family 2, member 4	Progressively increasing insulin resistance
	*PC-1*	Plasma cell membrane glycoprotein 1	Increased insulin resistance

## Data Availability

Not applicable.
